# Sexual Debut, Sexual Education, Abortion, Awareness and Prevalence of Contraceptive Among Female Undergraduates Students in Public and Private Universities in Ekiti State, Nigeria

**DOI:** 10.7759/cureus.28237

**Published:** 2022-08-21

**Authors:** Taofeek A Sanni, Olusegun E Elegbede, Kabir A Durowade, Kayode Adewoye, Tope M Ipinnimo, Ayo K Alabi, Austine Ibikunle, Olanrewaju K Olasehinde, Taiye A Alao, Olawale B Oni

**Affiliations:** 1 Department of Community Medicine, Afe Babalola University, Ado-Ekiti, NGA; 2 Department of Community Medicine, Federal Teaching Hospital Ido-Ekiti, Ido-Ekiti, NGA; 3 Department of Community Medicine, Afe Babalola University, Ado Ekiti, NGA; 4 Deprtment of Community Medicine, Federal Teaching Hospital Ido-Ekiti, Ido-Ekiti, NGA; 5 Department of Community Medicine, Federal Teaching Hospital, Ido-Ekiti, Ido Ekiti, NGA; 6 Public Health, Ogun State Hospital Management Board, Abeokuta, NGA

**Keywords:** abortion 2022, ekiti state, students, contraception, abortion, sexual debut

## Abstract

Introduction

Of the 182 million annual pregnancies in developing countries, 76 million are unintended and 66% of these are among non-users of contraception. Unintended pregnancy is a risk factor for abortion, disruption of education, future unemployment, and poor socio-economic status. This study aimed to determine the age of sexual debut, sexual education, abortion, awareness, and prevalence of contraceptives among female undergraduate students in public and private universities in Ekiti State.

Methods

A comparative cross-sectional study was carried out among 418 [public (208) and private (210)] female university undergraduate students in Ekiti State using a multi-stage sampling technique. Data was gathered using a semi-structured questionnaire and analyzed using SPSS (IBM Corp. Released 2015. IBM SPSS Statistics for Windows, Version 23.0. Armonk, NY: IBM Corp). Chi-square was used to assess the association between dependent and independent variables at the bivariate level of analysis. P-value<0.05 was taken as significant.

Results

The mean age of respondents was 21.1±2.5 years in the public univeristy and 19.3±2.1 years in the private university. About 53.8% of students in the public university have been engaged in sexual intercourse as against 30% of students in the private university. The mean age at first sexual debut was lower in the public university (14.2±4.1 years) than in the private university (16.9±3.3 years) while more public university students (87.5%) had access to sexual education than their counterparts at the private university (79.0%). Of those who had ever been pregnant in public university (18.8%), about four-fifth (81.1%) of them had an abortion while all those who ever got pregnant (15.9%) in private university had an abortion.

All the respondents in both universities were aware of contraception with the majority getting to know through social media. The prevalence of contraceptive use was lower among public university students (39.3%) than those in the private university (60.3%).

Conclusion

Mean age at sexual debut and rate of abortion were lower in public university students than in private. While access to sexual education was higher in the public university than in the private university, the prevalence of contraceptive use was lower in the public university.

Therefore it is recommended that the government and other relevant stakeholders need to institute continuous awareness campaign programs to increase contraception uptake and reduce the prevalence and effect of unwanted pregnancy as a result of unprotected sexual activities.

## Introduction

Of the 182 million annual pregnancies in developing countries, 76 million are unintended and 66% of these are among non-users of contraception [1]. Two-thirds of unsafe abortions occur among women between 15 and 30 years old and almost 14% of unsafe abortions in developing countries occur among women aged 20 years or below [2,3]. Unintended pregnancy is a risk factor for abortion, disruption of education, future unemployment, and poor socio-economic status [2,3].

Young people generally are sexually active, and with peer influence, the use of alcohol, and other illegal substitutes, the risk of unintended pregnancies is relatively high [4-6].

The Nigeria Demographic Health Survey, 2018 reveals that 19% of women begin sexual activity before the age of 15 years while more than half (57%) begin before the age of 18 [7]. It also reported the median age of first sexual intercourse as 17.2 years and 21.7 years for females and males respectively [7]. Early sexual debut (having first sexual intercourse at or before the age of 14 years) increases the risk of exposure to multiple sexual partners, unprotected sex, risk of sexually transmitted infections including HIV/AIDS, unwanted and teenage pregnancies, unsafe abortion just to mention a few [6].

In 2012 alone it was estimated that the unintended pregnancy rate in Nigeria was 56 per 1000 women aged 15-49 and 56% of these were resolved with abortion. The report also documented the rate of abortion in Nigeria as 33 per 1000 in women aged 15-49 [8]. Among university students, abortion appears relatively high with a rate of 60% globally according to an international study, 48% in Ghana, and 47.2% among university students in southeast Nigeria [9-11]. Though a low rate of 6.7% was recorded in northern Nigeria about 12.6% of the students still noted that induced abortion is a good way of resolving the challenge of unwanted pregnancy [12].

Abortion contributes about 40% of all maternal deaths in Nigeria [1,10,12]. This high mortality rate from abortion is due to the fact that 56% of abortions in the developing world are unsafe due to the restrictive law on abortion among other factors as compared to 6% in the developed world with liberal abortion laws [12-15]. Abortion constitutes an economic drain on the Nigerian health system and extra expenses on women for those who develop pelvic inflammatory diseases (PID) or infertility among other complications [16].

The use of contraceptive methods (a method or device used for the prevention of pregnancies) is required to control of number, timing, and spacing of births and prevention of unintended pregnancies [17-21]. Contraception use among women of 15-24 years is low especially with early exposure of a large number to sexual intercourse before marriage. According to a survey conducted among American and Canadian students aged 15-24 years, the prevalence of contraceptive use at first intercourse ranges from 4%-43% [22]. In Africa, 55% of women in the reproductive age group have an unmet need for modern contraception [17] while among unmarried youth in the west and central Africa the unmet need is about 41.7%. Despite this high unmet need and risky sexual intercourse, adequate health education and contraceptive use remain low in both first and last sexual encounters [21,23,24].

Comprehensive sex education and access to contraception have decreased the rate of unintended pregnancies, especially among the young age group [24,25]. Therefore, access to reproductive health education and youth-friendly services will help reduce the effect of inadequate knowledge and myth related to the side effects of contraception and enable young adults to make informed decisions about their reproductive health [26,27].

This study is aimed at determining the age of sexual debut, sexual education, abortion, awareness, and prevalence of contraceptives among female undergraduate students in public and private universities in Ekiti State, Nigeria

## Materials and methods

This study was carried out in Ekiti State, Nigeria. The State was created in October 1996 with its administrative headquarters located in Ado-Ekiti. Ekiti State has 16 local government areas (LGAs). It lies south of Kwara and Kogi States, and east of Osun State, while it is bounded in the east and in the south by Ondo State. Ekiti state has three senatorial districts: Ekiti Central, Ekiti South and Ekiti North senatorial districts. Ekiti Central and Ekiti North have five local government areas, while Ekiti South has six local government areas.

This study was a comparative cross-sectional study carried out among female undergraduate students in public (Federal University Oye) and private (Afe Babalola University Ado-Ekiti) universities in Ekiti State. Sample size was calculated using the formula for comparison of two proportions [28]. A total of 418 university undergraduate students in Ekiti State participated in the study (208 from public and 210 from private) which was carried out between November 2019 and January 2020 using a self-administered semi-structured questionnaire (adapted from the World Health Organization (WHO) questionnaire on reproductive health, population reference bureau questionnaire on contraception, birth control questionnaire of the University of Mary Washington student health centre and surveys on public knowledge and attitude on contraceptive and unplanned pregnancy).

A multistage sampling technique was used to select the eligible students for this study. In the first stage, Afe Babalola was purposively selected being the only private university in Ekiti State while Federal University Oye was selected using simple random sampling by balloting against Ekiti State University. At stage two, three faculties/colleges were selected by simple random sampling by balloting from each of the universities. In the third stage, three departments were selected by simple random sampling from the list of departments in the three colleges earlier selected in the first stage giving rise to nine departments per institution and questionnaires allocated equally to each department. And at the last stage, a systematic sampling technique was used after ascertaining the sampling interval via division of the sampling frame by the number of allocated questionnaires (25). The index respondent was selected using simple random sampling by balloting. Where selected respondents declined participation, a replacement was done by picking the next person on the list and subsequent application of sample interval from the picked respondents.

Data were analyzed using SPSS (IBM Corp. Released 2015. IBM SPSS Statistics for Windows, Version 23.0. Armonk, NY: IBM Corp). Categorical variables (e.g. religion, ethnicity, etc) were summarized as tables, proportions, and charts. Continuous variables (e.g. age) were summarized as means (standard deviation) and compared between the public and private universities. The Chi-square test was used to determine the statistical significance of observed differences in access to sexual education, sexual debut, abortion rate, awareness, and prevalence of contraception between public and private undergraduate students at the level of cross-tabulated variables. The level of significance was predetermined at a p-value of less than 0.05 at a 95% confidence level.

Ethical clearance for this study (Ref Number: ERC/2018/09/14/142A) was obtained from the Health Research and Ethical Committees of the Federal Teaching Hospital, Ido Ekiti. Permission was also sought from the university authority to conduct the research among undergraduate students of the university and informed consent was obtained from the respondents. Confidentiality was ensured through the anonymous distribution of the questionnaires.

## Results

Socio-demographic characteristics

The socio-demographic characteristics have been presented in Table 1. The mean age of female undergraduate university students in the public university was 21.1 ± 2.5 years while the mean age of female undergraduate students in the private university was 19.3± 2.1 years. There was a significant difference in the mean age of students in the public and private universities (p= <0.001). The majority of the students were single in both public (94.2%) and private (96.7%) universities; however, the number of married students in the public university (5.8%) was higher than that of private with only (3.3%). There but there was no difference in the marital status of the students in the two universities (p= 0.232).

**Table 1:** Socio-demographic Characteristics

Variable	Public n = 208 n (%)	Private n = 210 n (%)	Chi square	p-value
Age of respondent (in years)				
<20	63 (30.0)	121 (57.6)	35.812	<0.001*
20 – 24	124 (59.6)	84 (40.0)		
25 – 29	21 (10.1)	5 (2.4)		
Mean age ± SD	21.1 ± 2.5	19.3 ± 2.1	7.861^t^	<0.001*
Age Range	16 – 28	15 – 28		
Level				
100	30 (14.4)	39 (18.6)	7.350	0.119
200	41 (19.7)	23 (11.0)		
300	59 (28.4)	69 (32.9)		
400	47 (22.6)	44 (21.0)		
500	31 (14.9)	35 (16.7)		
Marital Status				
Married	12 (5.8)	7 (3.3)	1.429	0.232
Single	196 (94.2)	203 (96.7)		
Ethnicity				
Yoruba	170 (81.7)	118 (56.2)	41.771	<0.001*
Hausa	14 (6.7)	18 (8.6)		
Igbo	21 (10.1)	40 (19.0)		
Others	3 (1.5)	34 (16.2)		

A significant majority of the students were Yoruba (81.4%) in the public university followed by Igbo (10.1%) and Hausa (6.7%). However, only just above half (56.2%) were Yoruba in the private university, followed also by Igbo (19.0%), Hausa (8.6%) while others include Ibira, Tivi, Fulani, and others (16.2%). There was a statistically significant difference in the tribe (Yoruba) of the students in the public and private university and this was significant at p= <0.001.

Access to sexual education, sexual debut, and rate of abortion among female undergraduate students in public and private universities in Ekiti State

Table 2 shows the access to sexual education, sexual debut, and rate of abortion among female undergraduate students in public and private universities in Ekiti State. The majority of university students in both public (87.5%) and private (79.0%) had access to sex education with a difference in the two groups which was significant at p= 0.021. Close to half of these students in the public university had their education at home (41.2%), followed by seminars (37.9%), internet, and social media (34.6%) then health professionals (24.2%). Though the majority in private university also got their sex education at home (50.0%), then followed by seminars (44.6%), health professionals (41.0%), friends and peer groups (35.5%), TV, Radio, and newspaper (34.9%), only a few got their sex education through the internet and social media (6.7%).

**Table 2:** Sexual Education, Sexual Debut and Abortion Rate of Respondents

Variable	Public n = 208 n (%)	Private n = 210 n (%)	Chi square	p-value
Ever had access to formal sex education				
Yes	182 (87.5)	166 (79.0)	5.355	0.021
No	26 (12.5)	44 (21.0)		
Where, if YES*	n_1_ = 182	n_1_ = 166		
Internet and social media	63 (34.6)	11 (6.7)	36.122	<0.001
Home	75 (41.2)	83 (50.0)	2.707	0.100
Friends and peer	34 (18.7)	59 (35.5)	12.604	<0.001
Health professionals	44 (24.2)	68 (41.0)	11.211	0.001
Seminar	69 (37.9)	74 (44.6)	1.594	0.207
Religious Home	36 (19.8)	28 (16.9)	0.491	0.484
TV, Radio and Newspaper	29 (15.9)	58 (34.9)	16.725	<0.001
Others	29 (15.9)	58 (34.9)	16.725	<0.001
Ever had sexual intercourse				
Yes	112 (53.8)	63 (30.0)	24.415	<0.001
No	96 (46.2)	146 (70.0)		
Age at sexual debut (in years)	n_2_ = 112	n_2_ = 63		
< 9	18 (16.1)	3 (4.8)	26.632	<0.001
10 – 14	40 (35.7)	6 (9.5)		
15 – 19	37 (33.0)	44 (69.8)		
20 – 24	17 (15.2)	10 (15.9)		
Mean age at sexual debut ± SD	14.2±4.1	16.9±3.3	4.469^t^	<0.001
Age Range	7 – 22	5 – 22		
Ever been pregnant	n_2_ = 112	n_2_ = 63		
Yes	21 (18.8)	10 (15.9)	0.229	0.632
No	91 (81.2)	53 (84.1)		
Ever had an abortion before	n_3_ = 21	n_3_ = 10		
Yes	17 (81.0)	10 (100.0)	2.187	0.139
No	4 (19.0)	0 (0.0)		

More than half (53.8%) of the public university students had engaged in sexual intercourse at one time or the other as against less than one-third (30.0%) in the private university, and the difference in sexual exposure was significant (p= <0.001). For those who were sexually exposed in both institutions. The mean age of sexual debut among public university students was 14.2 ± 4.1 years while that of private was 16.9 ± 3.3 years. The difference in the mean age of sexual debut in the two groups was significant at p= <0.001

A sizable number of students (18.8%) in the public university and 15.9% in the private university had been pregnant before with 81% of them in public and 100% of them in private procuring abortion. There was no significant difference in the abortion rate between the two groups (p values 0.139).

Awareness of contraception among female undergraduate students in public and private universities in Ekiti State

Table 3 shows the awareness of contraception among female undergraduate students in public and private universities in Ekiti State. All the respondents in both public and private universities are aware of contraception. Sources were social media (more than one-third in public and more than two-third in private), friends (more than one quarter in public and more than half in private), mothers (more than one quarter in public and more than one-third in private), health care practitioners (more than one third in public and more than one quarter in private) and mass media (13.9% in public university students and 32.9% in the public university students). There were differences in sources of information from social media and internet (p= <0.001), friends (p= <0.001), mothers (p= <0.045), mass media (p= <0.001) and books/medical journals (p= <0.001) in the public and private students.

**Table 3:** Respondents’ Levels of Awareness about Contraception

Variable	Public n = 208 n (%)	Private n = 210 n (%)	Chi square	p-value
Awareness of Contraceptive				
Yes	208 (100.0)	210 (100.0)	*	*
Source(s) of Information*				
Healthcare Practitioner	69 (33.2)	59 (28.1)	1.268	0.260
Health Facility	26 (12.5)	33 (15.7)	1.158	0.282
Pharmacy Shop	29 (13.9)	38 (18.1)	1.339	0.247
Social Media and Internet	78 (37.5)	145 (69.0)	41.788	<0.001
Friends	57 (27.4)	110 (52.4)	27.174	<0.001
From mother	60 (28.8)	80 (38.1)	4.013	0.045
TV, Radio, Newspaper (Mass media)	29 (13.9)	69 (32.9)	20.830	<0.001
Books & Medical Journals	8 (3.8)	31 (14.8)	14.718	<0.001
Known type(s) *				
Barrier methods	208 (100.0)	210 (100.0)	*	*
Emergency Contraception	44 ((21.1)	86 (41.0)	19.116	<0.001
Combined Oral Contraception	22 (10.6)	96 (45.7)	63.679	<0.001
Progesterone Only Pills	58 (27.9)	97 (46.2)	15.009	<0.001
Injectables	63 (30.3)	52 (24.8)	1.600	0.206
Implants	34 (16.3)	89 (42.4)	34.107	<0.001
Intrauterine Devices	21 (10.1)	42 (20.0)	8.008	0.005
Withdrawal Method	77 (37.0)	105 (50.0)	7.163	0.007
Natural Family Planning Methods	72 (34.6)	96 (45.7)	5.355	0.021
Vasectomy	0 (0.0)	2 (1.0)	100.464	<0.001
Bilateral Tubal Ligation	1 (0.5)	10 (4.8)	7.475	0.006

Prevalence of Contraception among Female Undergraduate Students in Public and Private Universities in Ekiti State

Table 4 shows the prevalence of contraception among female undergraduate students in public and private universities in Ekiti State. The cumulative prevalence of contraceptive use among sexually exposed university students was 39.3% in public and 60.3% in private. The value of public university students was low compared to private. There was a significant difference in the prevalence of contraceptive use at p = 0.007.

**Table 4:** Prevalence of Contraception compared between Sexually Active Respondents

Variable	Public n = 112 n (%)	Private n = 63 n (%)	Chi square	p-value
Ever used any form of contraceptive				
Yes	44 (39.3)	38 (60.3)	7.162	0.007
No	68 (60.7)	25 (39.7)		

## Discussion

The mean age of female undergraduate students in this study was 21.1±2.5 years for the public university and 19.3±2.1 years for the private university. The mean age in this study is higher for public university undergraduate students than that of the private university though both still fall within the age range of university students in Nigeria. This difference in mean age in both settings is statistically significant at p<0.001. The higher mean age in public university students may mean higher sexual exposure, and a higher need for contraception and may also mean higher exposure and knowledge of contraception which may aid uptake. The findings in this study for a public university are similar to the mean age reported in studies done in Uganda to assess contraceptive use, knowledge, and sexual behavior of female university students where a mean age of 21.7 years was recorded and the majority (87.5%) of students fell between the age range of 20-24 years and another on long-acting reversible contraception (LARC) utilization among college students in Debre Berhan, Ethiopia where most (72.4%) of the respondents were within the age range 20-24 years [2,29].

The mean age of sexual debut in this study among public university students (14.2 ± 4.1) is lower than the private (16.9 ± 3.3). Also, the majority of the female students in public have their first sexual debut between the age range of 10-14 years while the majority of female students in private are within the age range of 15-19 years when they had their first sexual experience. This difference in the age of sexual debut is statistically significant at p<0.001. This may be due to better home training, discipline, and monitoring of the students in the private university. The mean value for the public university is lower than the national value of 15 years according to Demographic Health Survey (DHS) 2019 and this may increase the risk of unwanted pregnancies, sexually transmitted infections, and abortion [7]. However, the mean values from both public and private university students in this study are higher and better than finding among secondary school students in Ido-Ekiti, Ekiti State Nigeria with a mean age of sexual debut of 13.10 ± 2.83 years [12]. The age range for sexual debut in this study for public university students is lower than found among tertiary institution female students in Kano State Nigeria which documented a sexual debut age range of 16-20 years [30].

This study revealed that 87.5% (public) and 79% (private) of female undergraduate university students have received formal sex education one way or the other. This shows that female students in both universities have good exposure to sex education though slightly higher in public than in private. This difference is however not statistically significant. Means of education is majorly from the home, seminars, the internet, social media, health professionals, friend, and peer groups in both public and private university students. This is similar to the study done in Osun State among tertiary institution students where about 72.1% of them have received sexual education and another study done on LARC among adolescents and young women where 87% had attended sexual health clinics for sexual education [10,29]. Good sexual education has been documented to help reduce the occurrence of unwanted pregnancies and the use of contraception [7].

This study found that the occurrence of pregnancy in both universities is close with about (18.8% of public university students and 15.9% of private university students) being ever pregnant. While 81% of those pregnant in the public university had an abortion to terminate the pregnancy and others (19%) delivered the baby, all private university students who were pregnant terminated the pregnancy via abortion. The high abortion rate may be due to the negative effect of unwanted pregnancy, childbirth, and care of the baby can have on the student’s academic pursuits, freedom, access to abortion services, and also the societal perspective on being pregnant and having children before marriage. The difference in pregnancy and abortion rates between the two universities is not statistically significant. The abortion rate is similar to the 99% recorded in Kaduna State Nigeria but higher than the 40% reported in Uganda [1,29].

This study showed that all the respondents (100%) in both public and private universities have heard about contraception before. This is expected among the student groups with higher exposure to many educative materials and media. This finding is similar to studies done in Uganda among female undergraduates with an awareness rate of 99.6%, female university students in Lesotho with a rate of 97.5%, Ethiopia among female college students with a rate of 85.9%, and among college students in India 86% rate [2,5,26,29]. The sources of information in this study (both public and private) are majorly social media, mass media, friends and peers, mothers, healthcare practitioners, and books/journals. These findings are in keeping with findings among female university students in Lesotho and Kano Nigeria [26,30].

The general prevalence of contraception use in this study among students is low (39.3%) in the public university but slightly higher (60.3%) in the private university. There is a significant difference in this general prevalence between the public and private and this might be due to poor attitude to contraceptive usage in the public students. The prevalence of contraception in the public university is considerably low in relation to the level of sexual exposure in the university (53.8% are sexually exposed and about 50% in having sexual relationships). This study finding is lower in the public university but higher in private than the 46% of contraceptive use reported among female undergraduate university students in Uganda [29]. However, both are higher and well above the 15.63% reported among female unmarried tertiary institution students in Kano State Nigeria [30].

## Conclusions

The mean age at first sexual debut was lower in public university students than in private and this difference is statistically significant (p<0.001). Also, the rate of abortion is generally high though was lower among public university students than in private but the difference is not statistically significant. Access to sexual education was higher in the public university than in the private university and this difference is statistically significant (p=0.021). While awareness about contraception was 100% in both universities, the prevalence was lower in the public university than in private with the difference being statistically significant (p=0.007).

Government and other relevant stakeholders need to institute continuous awareness campaign programs to increase the uptake of contraception in order to reduce the prevalence and effect of unwanted pregnancy as a result of unprotected sexual activities.
